# Research progress on microbial adsorption of radioactive nuclides in deep geological environments

**DOI:** 10.3389/fmicb.2024.1430498

**Published:** 2024-07-03

**Authors:** Tianyu Wang, Qichao Zhang, Yanxin Qiao, Yishan Jiang, Feng Xiao, Jizhou Duan, Xin Zhao

**Affiliations:** ^1^Navy Submarine Academy, Qingdao, China; ^2^School of Materials Science and Engineering, Jiangsu University of Science and Technology, Zhenjiang, China; ^3^CAS Key Laboratory of Marine Environment of Corrosion and Bio-Fouling, Institute of Oceanology, Chinese Academy of Sciences, Qingdao, China

**Keywords:** microorganism, radioactive nuclides, nuclear waste, microbial adsorption, deep geological environments

## Abstract

Due to the development and utilization of nuclear energy, the safe disposal of nuclear waste needs to be urgently addressed. In recent years, the utilization of microorganisms’ adsorption capacity to dispose of radioactive waste has received increasing attention. When compared with conventional disposal methods, microbial adsorption exhibits the characteristics of high efficiency, low cost, and no secondary pollution. In the long term, microbial biomass shows significant promise as specific chemical-binding agents. Optimization of biosorption conditions, identification of rare earth element binding sites, and studies on the sorption capacities of immobilized cells provide compelling reasons to consider biosorption for industrial applications in heavy metal removal from solutions. However, the interaction mechanism between microorganisms and radioactive nuclides is very complex. This mini-review briefly provides an overview of the preparation methods, factors affecting the adsorption capacity, and the mechanisms of microbial adsorbents.

## 1 Introduction

As a new generation of efficient energy, nuclear energy has the characteristics of low cost, small size, large heat, convenient transportation and cleanliness. However, with the rapid development of the nuclear energy industry, the problem of safe disposal of nuclear waste needs to be solved urgently. The nuclear waste mainly includes U, Am, Cs, Sr., Th and other radioactive nuclides, which usually exist in the form of ions and have strong migration ability. Its decay will cause radiation damage to organisms, and improper disposal will seriously threaten the living environment of human beings (Qiao et al., 2024). At present, the main dispose method for radioactive nuclides is solidification treatment, which is to fix the radioactive nuclides in a stable solid medium, so that the radioactive material can be transformed into a solidified body that is easy to transport, store and operate (Galamboš et al., 2011; Ighalo et al., 2024). The solidified body is then packed into a metal disposal container and buried deep in an underground repository, allowing it to decay naturally and be permanently isolated from the human living environment. However, the presence of aggressive anions in the underground repository environment can cause corrosion of the metal containers, which in severe cases can lead to failure and eventual leakage of the nuclear waste inside.

As early as the 1940s, it was observed that some microorganisms have a special affinity for metal ions (Rushhoft, 1949). Since then, the research on the recovery of precious metals by microorganisms has attracted the attention of foreign scholars and gradually entered the practical industrial application. Adsorption is regarded as one of the most efficient methods for removing U (VI) from wastewater due to its high efficiency, affordability, and simplicity (Zein et al., 2023; Ahmadi et al., 2024). It also abides by the ideals of sustainable development and green chemistry and has rapid adsorption kinetics, a large amount of adsorption capacity, and strong adsorption–desorption performance (Meng et al., 2019; Ramli et al., 2023). Through the adsorption, transformation and fixation of radioactive nuclides by microorganisms, the original curing method has been broken through to a certain extent. This method has the characteristics of low investment, low energy consumption and high efficiency, no secondary pollution, and can even effectively recycle some nuclides (Kim and Park, 2001; Dong et al., 2004; Kleinübing et al., 2011). In nature, microorganisms are not only widely distributed and diverse, but also multiply quickly, have small size, large specific surface area and have strong adaptability. There are dozens of microbial species that adsorb and enrich radioactive nuclides, mainly including bacteria, fungi and algae.

## 2 Preparations of biosorbent

Excellent stability, low toxicity, great selectivity, and high affinity are requirements for effective adsorbents. The secret to enhancing adsorption performance is to increase the adsorbent’s adsorption capacity through some kind of preparation technique of modification/treatment (Umeh et al., 2023; Ighalo, 2024). After selecting a suitable microbial producer, it is also necessary to predispose the microorganisms to improve their adaptability to the environment and make them have a better adsorption effect on radioactive nuclides. At present, the main methods used include: chemical method, physical method, immobilization, inorganic salt activation, etc., or modern technological means such as protein modification and genetic modification are used to dispose cells at the molecular level (Choi and Yun, 2004). The main purpose of which is to deprotonate the surface of the adsorbent and activate the adsorption site, so as to improve the chemical properties of the adsorbent. The summary of the preparations of biosorbent is shown in Table 1, which introduce some methods and effects about different preparations of biosorbents.

**Table 1:** Summary of the preparations of biosorbent.

Preperations	Methods	Effects
Physical and chemical methods	Chemical methods(NaOH, HNO3, et al.) Physical methods(dry, grind, radiate, et al.)	The adsorption capacity of *S. cerevisiae* disposed with NaOH increased by 5 ~ 30% HNO_3_ did not significantly improve its adsorption capacity The Sr. removal rate of disposed *S. cerevisiae* reached 95%
Immobilize microorganism technology	Physical adsorption Embedding in agar Embedding in sodium alginate-gelatin Embedding in polyacrylamide gel	Improve mechanical strength, chemical stability, physical form, mass transfer permeability, resistance to degradation Reuse in addition to high adsorption rate and large adsorption capacity.
Genetic engineering	Create radiation-resistant microorganisms (*D. radiodurans*, et al.) Recombinant genetic engineering (constructe *D. radiodurans* containing phoN gene encoding nonspecific acid phosphatase) Radiation resistance gene: recA Damage-repair gene: pprA	*D. radiodurans* is extremely tolerant to ionizing radiation, ultraviolet radiation, et al. The new E-coli using pprA gene had better oxidation resistance *D. radiodurans*’ recA can make DNA repair faster and more efficient

### 2.1 Physical and chemical methods

Chemical pretreatment can enhance the selectivity and adsorption efficiency of the biosorbents. All biological moieties including bacteria, fungi, algae, plants, and animal biomass, as well as derived products such as chitosan, have the capability of microbial adsorption. Specific functional groups present on the cell surface, such as carboxyl, amine, hydroxyl, phosphate, and sulfhydryl groups, interact with the metal and lead to the sorption of metals (Banala et al., 2021). Alkali dispose can increase the adsorption site of the adsorbent and expose additional functional groups (Brierley, 1990). The system can also be alkalized, and the buffer media can no longer be added in subsequent steps, thus simplifying the process and making it easy to industrialize. Liao et al. compared the U (VI) adsorption capacities of a PMBC-based adsorbent. They discovered that pretreatment with potassium permanganate significantly increased the capacity of biochar to adsorb U(VI) (979.3 mg/g) by activating many groups on the material’s surface (Liao et al., 2022). Xiong et al. (2022) used HAP aerogels with strong chemical bonding between phosphate and U(VI) to maximize the adsorption capacity, achieving an adsorption efficiency of 99.4% for U(VI) at pH 4.0 and 25°C, with an adsorption capacity of 2087.6 mg/g. Juan and Liyu (1999) showed that the adsorption capacity of white-rot-fungus disposed with 0.1 mol/L NaOH for Pb increased significantly, reaching 23.66 mg/g. They believe the reason is that NaOH can dissolve impurities on the cell that are not conducive to adsorption and neutralize H^+^, exposing more active binding sites on the cell and increasing the adsorption capacity. *P. laminosum* Sampedro et al. (1995) disposed *S. cerevisiae* with 1 mol/L HNO_3_ did not significantly improve its adsorption capacity, while disposed with 1 mol/L NaOH, the adsorption capacity of *S. cerevisiae* increased by 5 ~ 30%.

Physical pretreatment refers to a variety of physical ways to make microorganisms produce changes conducive to adsorption, including drying, grinding, radiation and other methods. Domesticated under Sr.(II) stress promoted the microbial adsorption ability of *B. pumilus* to Sr.(II), and the microbial adsorption efficiency increased from 46.09 to 94.69%. At a lower initial concentration, the living bacteria had the ability to resist the microbial adsorption of Sr.(II) (Dai et al., 2020). Suslov et al. (2004) isolated a series of *S. cerevisiae* from extreme environments, cultured the *S. cerevisiae* on 0.1 mol/L Sr. medium, and found that its accumulation efficiency of Sr. could reach 80%. Mingxue (2011) used Sr. for stress induced domestication after radiation pretreatment of *S. cerevisiae*, and found that the Sr. removal rate of disposed *S. cerevisiae* reached 95% in simulated low-level waste solution. They used the accelerator to domesticate and select 8 strains of *S. cerevisiae* with high tolerance to U and Sr. by pulsed X-ray irradiation and stress induction. The results indicated that *S. cerevisiae* cells can tolerate irradiation with high doses of pulsed X-rays. After domestication, they have strong tolerance to U and Sr., and the adsorption rate of U and Sr. can reach 90% (Liu et al., 2016).

### 2.2 Immobilize microorganism technology

The disadvantages of free and suspended microorganisms are fine particle and low mechanical strength, which can be overcome by solidified cells. Immobilize biotechnology refers to the process of solidifying cells in a free state by physical or chemical methods. At present, the main methods used are physical adsorption, embedding in agar, embedding in sodium alginate-gelatin and embedding in polyacrylamide gel. Compared with the conventional methods of microbial adsorption of radioactive nuclides, solidified microorganisms have advantages in mechanical strength, chemical stability, physical form, mass transfer permeability, resistance to degradation, and reuse in addition to high adsorption rate and large adsorption capacity. At present, solidified microorganisms are mainly applied to dispose the wastewater contaminated by heavy metals and organic matter, while relatively little research has been done on disposing radioactive waste (Xueqin et al., 2006; Yujian and Hongyu, 2006).

**Figure 1:**
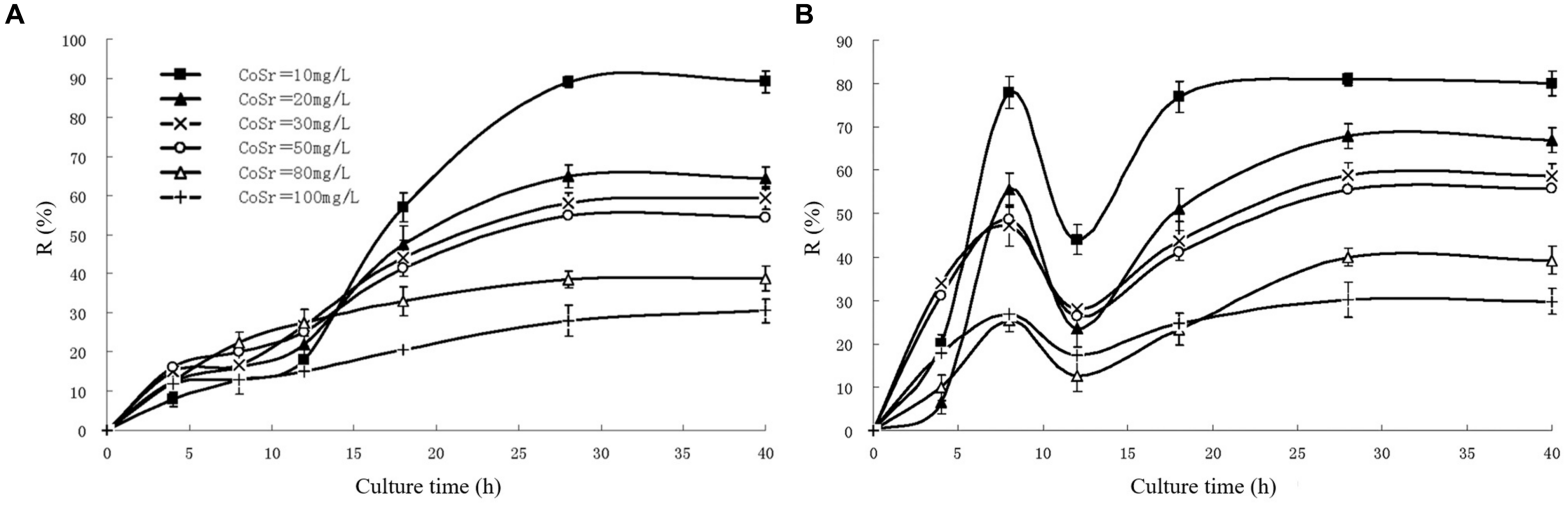
The adsorption rate of strontium by cerevisia particles **(A)** unsolidified **(B)** solidified (Mingxue, 2011).

As shown in Figure 1A, the adsorption rate of uncured *S. cerevisiae* decreased with the increase of strontium concentration, and the adsorption rate was close to 90% when the strontium concentration was 10 mg/L. Figure 1B shows the curve of strontium adsorption rate of immobilized *S. cerevisiae* with time. The adsorption rate increased rapidly at the beginning and reached the maximum at 8 h, which was mainly because the adsorption of strontium ions by calcium alginate particles. After that, due to the shedding of surface particles, the adsorption rate decreased temporarily. As the *S. cerevisiae* began to participate in the adsorption, the whole process entered the second stage, and the adsorption equilibrium was reached after 20 h. Rome and Gadd (1991) used *S. cerevisiae* fixed in calcium alginate particles to carry out ion removal experiments. The results showed that the immobilized *S. cerevisiae* cells exhibited better adsorption effect than free cells.

Macaskie and Dean (1989) successfully fixed a strain of *Citrobacter* to remove radioactive U and Pb by using gel embedding method and glass spirochetes adsorption method. The results showed that the removal rates of U and Pb reached 99 and 94%, respectively. Akhtar et al. (2009) solidified *Trichoderma harzianum* with calcium alginate and found that the solidified *Trichoderma harzianum* had better stability. The adsorption column equipped with 1.5 g calcium alginate immobilized particles could purify 8.5 L of U solution (25 mg/L), and the adsorbed U could be eluted with 0.1 mol/L HNO3. The elution rate was 98.1% ~ 99.3%. The above results indicate that solidify microbial cells can improve their adsorption effect and play a better role in environmental pollution control.

### 2.3 Genetic engineering

In recent years, the use of gene modification to dispose microorganisms has attracted extensive attention from scholars. Because the radioactivity of radioactive nuclides may have an effect on the microorganism, and even lead to a decrease in its adsorption capacity. Currently, *D. radiodurans* is widely studied at home and abroad. Studies have shown that *D. radiodurans* is extremely tolerant to extreme environments such as ionizing radiation, ultraviolet radiation and oxidation resistance. And can avoid death or mutagenic changes under acute gamma irradiation exceeding 15 KGy. Moreover, it can grow continuously under chronic gamma irradiation (60Gy/h), and its growth rate and expression of cloned genes are not affected by irradiation (Jianlong, 2004).

In terms of construction and application of genetically engineered bacteria, Appukuttan et al. (2006) constructed a strain of *D. radiodurans* containing phoN gene encoding nonspecific acid phosphatase. The recombinant genetically engineered bacteria precipitated 90% of U from 0.8 mM acid solution within 6 h. RecA is one of the radiation-resistant genes cloned from *D. radiodurans*. Compared with *E. coli’s* recA, recA is more likely to bind DSBs (double-stranded DNA fragments), which can make DNA repair faster and more efficient. Other studies found that recA of E.coli could not compensate for the absence of recA of *D. radiodurans*, indicating that recA of *D. radiodurans* had a unique and extraordinary anti-radiation DNA repair function (Bonacossa de Almeida et al., 2002; Haruta et al., 2003). Disruption of recX (dr1310) in *D. radiodurans* using targeted mutagenesis method enhanced its ROS scavenging activity, and recX overexpression in this bacterium repressed its antioxidant activity significantly (Sheng et al., 2005).

Satoh et al. (2002) found a damage repair gene pprA in DR, and the study found that the deletion of this gene would lead to the decline of radiation resistance. The pprA protein has DNA-binding activity and plays an important role in non-homologous DNA recombination repair independent of recA pathway. At the same time, Kota and Misra (2006) constructed a new E-coli using pprA gene. Compared with ordinary *E. coli*, the newly constructed strain had better oxidation resistance.

## 3 Affected factors of microbial adsorption

The adsorption behavior of radioactive nuclides by microorganisms is influenced by numerous physical and chemical factors. It includes the physical and chemical properties of the biosorbent and the adsorbed ion itself. As well as various environmental conditions, such as pH, time, temperature, etc. Simultaneously, it is also impacted by the physiological conditions of the microorganisms themselves, including bacterial activity and concentration.

### 3.1 pH

The pH of the aqueous solution is the main factor affecting the saturated adsorption amount due to the competitive adsorption effect between H^+^ and the adsorbed cations. The competitive adsorption effect means that when systemic pH is low, H_3_O^+^ occupies a large number of adsorption active sites, preventing the cation from contacting with the adsorption active sites. As a result, the adsorption capacity decreases Many studies have shown that when the pH fluctuates within a certain range, the adsorption amount will change with the pH value. But it will not be a simple linear relationship.

The effectiveness of the adsorbent’s active sites is often diminished by cations with large radii and high valence states, which lowers the adsorbent’s efficiency for adsorbing U(VI) (Ding et al., 2021). HCP adsorbents containing carboxyl and amino groups, according to Bai et al. (2020), primarily rely on inner-sphere surface complexation rather than ion exchange, indicating that strong ionic strength has little bearing on uranium adsorption onto polymers. Brady et al. (1994) showed that the optimum pH for Cu^2+^ adsorption by *S. cerevisiae* was 5 ~ 9, and that adsorption of Cu^2+^ was reduced under any limiting conditions, especially at low pH. Dhami et al. (1998) used R. Arrhizus to adsorb radioactive nuclides, and found that the pH of Am, Ce, Pu, Eu and Zr reached 2.0 when the maximum adsorption capacity was reached. With the increase of pH, the adsorption capacity of U and Pu increases, and the maximum value is reached when the pH is 6 ~ 7. And then the adsorption decreased gradually when the pH continued to increase. Kazy et al. (2009) adsorbed U and Th with Pseudomonas, it was found that the maximum adsorption capacity reached 150 mg/L when the pH reached 4.0, because Th_2_(OH)_2_ and other polymers were formed to improve the adsorption capacity of element Th. Because U is a univalent polymer, UO_2_ will be formed when pH = 2.0, so that the adsorption capacity of U can reach a maximum of 400 mg/L.

### 3.2 Temperature

Temperature has an effect on the growth of microorganisms and can influence the adsorption of metals by affecting the metabolic activity of the microorganisms, the thermodynamic force of group adsorption and the heat capacity of adsorption. For active microorganisms, it is at the right temperature that their own metabolism can be optimized. However, for dead organisms, the effect of temperature is not significant. For some biosorbents, temperature is an important factor, but generally not as significant as pH. And the effect on different microorganisms or adsorbed metal ions is different.

In the adsorption of U and Th with R. Arrhizus Tsezos and Volesky (1981) found that the amount of U and Th adsorbed increased only slightly when the temperature was increased from 5°C to 40°C. In contrast, in the range between 2°C and 7°C, the adsorption of U by K. marxianus MB3 increased sharply with increasing temperature (Bustard et al., 1997). Some studies have also shown that the effect of temperature on adsorption is also related to pH and equilibrium concentration. Cossich et al. (2002) found that the adsorption capacity of *Sargassum* sp. for Cr at pH = 3.0 had a definite increase when the temperature was changed from 30°C to 40°C. At pH = 4.0 and high equilibrium concentrations, there was a significant increase in the adsorption capacity with the change in temperature, which did not occur at low equilibrium concentrations. In general, the effect of temperature on adsorption is not obvious. But in practical applications, considering the factors of operating costs and operating environment, it is also necessary to choose the appropriate temperature.

### 3.3 Time

Adsorption time is also an important factor that affecting the efficiency of metal adsorption by microorganisms. A long enough time is required during the adsorption process for the adsorption to reach equilibrium, thus effectively adsorbing or removing metal ions. Generally, it requires 2 ~ 4 h or longer to reach adsorption equilibrium. Scholars generally believe that the adsorption of metal ions by biomaterials can be divided into two stages. The first stage is the fast adsorption stage, which usually reaches about 70% of the final adsorption amount in a few minutes; the second stage is the slow adsorption stage, which often takes several hours or more to reach the final adsorption amount. The former is a rapid surface adsorption, while the latter often involves the transformation of metal ions to the cell, controlled by intracellular metabolism and cell diffusion processes (Ning, 2006).

Kazy et al. (2009) adsorbed U and Th with Pseudomonas and found that the adsorption equilibrium was reached within 2 ~ 4 h. The maximum adsorption was 500 mg/L and 170 mg/L, respectively. Nakajima and Tsuruta (2004) isolated *Lactobacillus* sp. from U mines can clear 2,200 μmol/g of U within 1 h. Khani et al. (2008) investigated the adsorption of *Catenella repens* at low pH and showed that *Catenella repens* could adsorb 90% of U within 30 min and reach equilibrium at 45 min with adsorption of up to 303 mg/g. Shunzhong et al. (2003) studied the adsorption of ^241^Am by R. Arrhizus and the results showed that the adsorption rate reached 69.7% at 15 min and 93.8% at 60 min, after which the adsorption rate increased slowly and reached an equilibrium of about 97% by 2 h.

### 3.4 Bacterial concentration

In practical application, the amount of microorganisms placed and its cost-effectiveness should also be taken into account while achieving the role of removing radioactive nuclides. Overseas researchers choose live and dead Pseudomonas to adsorb U and Th, and found that the adsorption rate is proportional to the concentration of the bacterium in the concentration of 0.2 ~ 3.0 mg/L. And the adsorption rate is 100% when the concentration of the bacterium reaches 3 mg (Andres et al., 1993). When studying the adsorption capacity of microorganisms to U Takehiko (2004) found that actinomycetes had the strongest ability, and the most prominent stem cells were Streptomyces levoris, which could adsorb U by 380 μmol/g.

In addition, it will also affect its adsorption capacity whether the microorganism is alive. There is a bioaccumulation process in the adsorption of living cells. However, in the actual adsorption process, the adsorption capacity of living cells is not higher than that of dead cells due to the participation of the energy metabolism system (Golab et al., 1991). Ling et al. (2001) studied the adsorption capacity of Cu^2+^, Pb^2+^, Ni^2+^, Zn^2+^, Ag^+^ and Cd^2+^ by living and dead organisms of *Prorocentrum micans*. The experiment showed that after 30 min adsorption of metal ion mixture by *Prorocentrum micans*, the concentration of each ion decreased significantly and reached equilibrium. The living and dead *Prorocentrum micans* have similar adsorption capacity for these six metal ions. The results showed that living *S. horneri* biomass was effective at scavenging Sr., Co, and Mn from seawater in both mono-and multi-nuclide contamination scenarios. Notably, the removal efficiency of *S. horneri* was found to be in the following order: Mn > Co > Sr (Wang et al., 2021).

### 3.5 Microorganisms in a radioactive environment

The presence of radionuclides results in the accumulation of significant amounts of radioactive materials within living organisms, leading to the degradation of cellular structures and vital biological molecules such as proteins and DNA. For instance, magnetic bacteria, which orient themselves by sensing the geomagnetic field, may suffer disrupted migration due to radioactive disturbances, thereby impacting the availability and ecological equilibrium of other organisms within the food chain. Consequently, organisms may experience impaired physiological functions, disrupted hormonal balance, reduced immunity, and potential reproductive failure and genetic mutations. Radioactive substances accumulate in the food chain in a cascading manner: initially contaminating microorganisms are ingested by herbivores and subsequently transferred to predators through predatory interactions.

However, less is known about microorganisms in the nuclear environment. An understanding of the microbial communities in the nuclear environment has been developed through microbial distribution studies at uranium mines, high and low volume liquid waste storage tanks, reactors, and nuclear waste repositories. It has been found that a large number of microorganisms, including bacteria and fungi, grow in the nuclear environment. Microbial taxa were analyzed in sediments from the leachate area of the United States Department of Energy’s Nuclear Waste Storage Site (NWSS) in Washington State. The sediments were contaminated by a waste tank constructed in 1962 to store high concentrations of alkali, nitric acid, aluminum, chromium, Cs-137, and Tc-99 waste streams. The study isolated viable microorganisms from 11 of the 16 samples, but cell counts were low, with a maximum of only 10^4^ clones forming units per gram of sample. *Arthrobacter* was the most abundant, with distribution in all samples. Other microorganisms with high G + C content such as *Rhodococcus* and Nocaedia were also present (Mingxue, 2011). In addition, Ruggiero et al. studied the effect of the toxicity of actinides, metal ions, etc. on *D. radiodurans* and *Pseudomonas putida*. It was found that Pu(IV), U(VI) and Np(V) inhibited the growth of *D. radiodurans* at concentrations of 5.2, 2.5 and 2.1 mM, respectively; and inhibited the growth of *Pseudomonas putida* at concentrations of.

Some microbes hold abilities to reduce/oxidize metals, such as U, Cr, Pb, etc. Lovley et al. (1991) first reported that the Fe (III)-reducing bacteria *G. metallireducens* and *S. oneidensis* could gain energy by reducing U(VI) for anaerobic growth, demonstrating that microorganisms are capable of heterogeneously reducing U(VI). On this basis, a large number of studies have utilized the reduction of microorganisms to immobilize radionuclides such as uranium *in situ*, thereby realizing the control of nuclide migration at uranium-contaminated sites.

## 4 Mechanisms of interaction between biosorbents and radioactive nuclides

**Figure 2:**
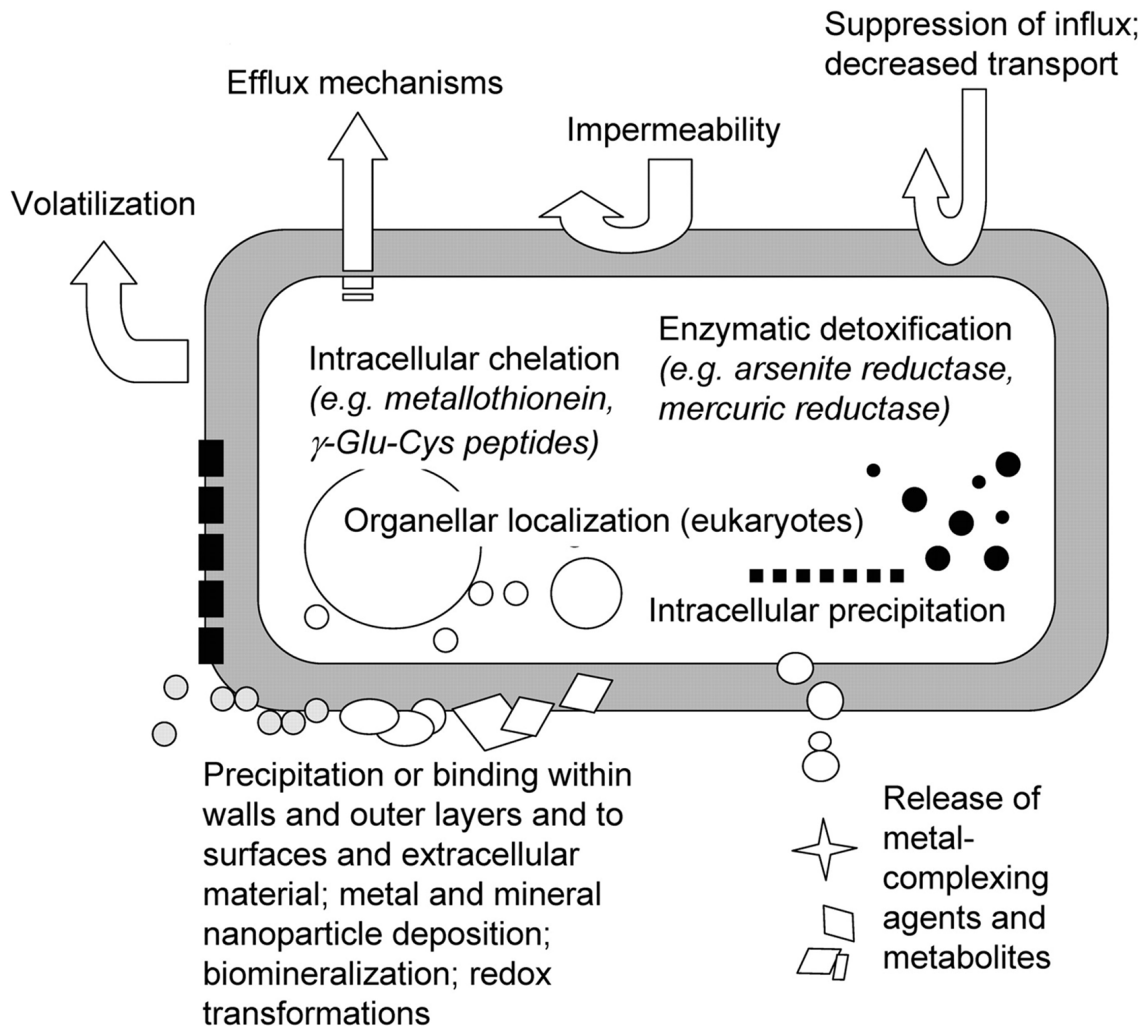
Mechanisms of microorganism adsorption (Gadd, 2010).

Figure 2 illustrates the interaction between microorganisms and radioactive nuclides. Bacterial resistance mechanisms include efflux and enzymatic detoxification, which have the potential to release intracellular toxic metals, e.g., Hg(II) reduction to Hg(0) (Osman and Cavet, 2008). Bacterial plasmids and chromosomes contain resistance genes to a variety of toxic metals, including Ag^+^, AsO_2_^−^, Cd^2+^, Co^2+^, CrO_4_^2−^, Cu^2+^, etc. The sequences of these genes have been determined and resistance mechanisms have been proposed. Metal concentrations in fungal cells are also affected by translocation regulation and compartmentalization, including exocytosis mechanisms and internal compartmentalization. Microorganisms also synthesize metal-binding peptides and proteins, such as metallothioneins and phytochelatins, which balance metal ions and influence toxic responses (Eide, 2000; Avery, 2001). In eukaryotes, intracellular partitioning also plays an important role in tolerance. The study of resistance in bacteria and fungi is important for understanding how microorganisms respond to toxic metals and other antibiotics in the environment.

Based on the diverse modes of interaction, the process of metal ion absorption by organisms can be primarily divided into two stages: (i) adsorption of metal ions on the cell surface: passive adsorption facilitated by extracellular polymers and functional groups on the cell wall to metal ions. It is characterized by rapid, reversible, and independent of energy metabolism (ii) active adsorption involving living cells: mainly encompasses the influence of substances generated during microbial metabolism and activities of biological macromolecules on nuclide adsorption, leading to intracellular accumulation. Theoretically, living cells should exhibit higher adsorption capacity than dead cells. However, due to high concentrations of heavy metal ions inhibiting biological cell metabolism, the second stage of living cell adsorption becomes sluggish. Consequently, in practical adsorption processes where energy metabolism systems are involved, the adsorption capacity of living cells does not surpass that of dead cells. As research progresses further in this field, it has been discovered that biological adsorption results from a combination of multiple mechanisms rather than a single mechanism alone. So far, the following adsorption mechanisms have been generally accepted by scholars at home and abroad.

### 4.1 Extracellular deposition

Popa et al. (2003) studied the adsorption of U by cerevisiae, they found that U attachments appeared on the surface of cerevisiae through SEM. During the process of adsorption of U by *Pseudomonas fluorescens*, it was also found that the complexes formed by U covered the whole surface of the cells and were a complex of membrane-peptidoglycan-plasmalemma, consisting of plate U mineralized particles with the shape of 10 nm ~ 1 μm. Strandberg et al. (1981) studied the adsorption of U by *S. cerevisiae* and *Pseudomonas aeruginosa* cells, they found that U was deposited on the cell surface in the shape of acicular fiber layer. At the same time, it was found that U entered the cell very fast (10s), which may have nothing to do with cell metabolism. Microorganisms can also precipitate nuclides by producing ligands such as PO_3_^3−^, SO_4_^2−^, CO_3_^2−^ and OH^−^. Microorganisms effectively remove the nuclides by producing a high concentration of ligands around the cell, thereby forming a precipitated nucleation site around the cell. Among them, the precipitation of nuclides by phosphate production by Serratia is the most widely studied (Pattanapipitpaisal et al., 2002). Glycerol-2-phosphate is produced by phospholipase hydrolysis to produce inorganic phosphate (Yong et al., 2004), which can form cell-bound metal phosphate with heavy metal ions, thus achieving the purpose of removing nuclides. Sheng et al. (2022) explores the extent to which interaction with primary silicates and secondary minerals affects the activity and longevity of a model soil enzyme β-glucosidase (BG). In Sheng et al.’s (2023) study, the bioavailability of mineral-bound Mo, V, and Fe was determined by incubating an obligately anaerobic diazotroph *Clostridium kluyveri* with Mo-, V-, and Fe-bearing minerals (molybdenite, cavansite, and ferrihydrite, respectively) and basalt under diazotrophic conditions. They found that as a result of microbial weathering, mineral surface chemistry significantly changed, likely due to surface coating by microbial exudates for metal extraction.

### 4.2 Extracellular complexation

The cell wall is also a major accumulation site for metal ions. The surface structure of bacterial cell wall is mainly composed of polysaccharides, protein and chitin. N, O, S atoms of hydroxy, carboxyl, phosphoryl and acylamino in the surface structure can be used as coordination atoms to coordinate with radionuclide ions. It has been reported that Zn and Pb can form complexes with phosphoryl and carboxyl groups on the surface of *P. chrysogenum*. Anions (EDTA, SO_4_^2−^, C1^−^, PO_4_^3−^, etc.) appearing in the solution can compete with cells to form complexes of heavy metal cations. Thereby reducing the adsorption amount of metal ions, which can also reflect the existence of the complexation mechanism from another aspect (Can and Jianlong, 2006). A few studies have shown that uranyl ions mainly interact with carboxyl and phosphoryl groups in adsorption process (Renshaw et al., 2007). Merroun et al. (2005) studied the adsorption of *Bacillus sphaericus* JG-A12 on uranyl ion. They found that U can interact with S-layer. XAS results show that uranyl ion forms a binary complex with carboxyl group and a unary complex with phosphoryl group. So that U accumulates in a dense way on the cell surface. *S. cerevisiae* exhibits good performance in the enrichment of uranium and strontium with as high as almost 90% microbial adsorption efficiency. The results demonstrate that adsorbed uranium and strontium precipitates can be transformed into authenite and strontium sulfate on cell surface. The final state of uranium is mainly in form of UP_2_O_7_, while the final state of Sr.(II) is mainly in form of SrSO_4_ after ashing (Zhou et al., 2022). Recently, marine yeast Yarrowia lipolytica has been explored for uranyl removal (Kolhe et al., 2020).

### 4.3 Intracellular enrichment

Since they studied the adsorption of Sr. by cerevisiae Avery and Tobin (1992) found that adding glucose could stimulate Sr. absorption, and they believed that glucose might promote the synthesis and activity of transporters on the cell membrane. Sr^2+^ internal flow and H^+^ outflow occurred when glucose was added 1 ~ 2 min later, and Sr^2+^ was enriched in the cellular vacuole and other locations. However more studies have found that intracellular accumulation is often unrelated to metabolism. For example Volesky and May-Phillips (1995) and Renshaw et al. (2007) found that U could accumulate both inside and outside cerevisiae cells and eventually form aciculr crystals. They believe that this process has nothing to do with metabolism and is mainly because the high osmotic pressure that U forms on the cell membrane. It is worth mentioning that some microorganisms do not die when they are enriched in U intracellularly. For example, *Arthrobacter ilicis*, a bacterium isolated from acidic U contaminated soil, can form U precipitation in the intracellularly, which is related to polyphosphate particles. Researchers believe that this intracellular precipitation is a detoxification mechanism of microorganisms (Yohey and Jillian, 2004). In addition, other studies have shown that the accumulation of Pu in cells is metabolically dependent, and this process depends on the iron transport system to form the U (IV)-desferrioxamine B complex (John et al., 2001).

## 5 Conclusion and outlook

Given the extensive research conducted in recent years, microorganisms have demonstrated significant potential as adsorbents. Moreover, the utilization of microorganisms for radionuclide treatment has gained widespread acceptance. In comparison to conventional solidification, this approach offers notable advantages in terms of both cost-effectiveness and efficiency, particularly when addressing nuclear wastewater. However, several challenges still need to be addressed, including:

1. Adsorption is a preferred technology for the removal of U(VI) from the aqueous phase. Super adsorbents have been shown to have an adsorption capacity of >1,000 mg/g. Additionally, the adsorbent can be degraded and recycled. If the source of the adsorbent is environmentally friendly, then it can also be considered an environmentally friendly process. The main challenge in this application is the scalability of the technology. To achieve high throughput, a column adsorption configuration is required, and the adsorbent must be able to be reused many times before it permanently loses its adsorption capacity. This is particularly important for expensive engineered adsorbent materials. We suggest that further investigation into adsorption studies be conducted. The radiation resistance and adsorption capacity of microorganisms cannot be simultaneously achieved, which is currently the most intractable problem;
2. Following the recovery of the adsorbent, it can be remediated using biological remediation techniques. However, biological remediation techniques is highly sensitive to pH and temperature, and therefore requires very close monitoring of parameters. Given that biological remediation techniques is relatively less efficient than other high-capacity processes, further research is needed in this area;
3. There is a possibility of process automation. The automation of the microorganism treatment process and the adsorption process can provide strong supporting conditions for microorganism adsorption to be implemented on a large scale;
4. Modern science and technology are being applied. For example, the adsorption mechanism has been further studied by means of FTIR spectroscopy and isotope tracing methods. Additionally, genetic engineering technology is used to achieve precise transformation of microorganisms.

Furthermore, it was observed that membrane filtration and ion exchange are the most promising processes for this application. Membrane processes have the advantage of high throughput, although they do present a challenge in terms of fouling. In addition to its high pH sensitivity, ion exchange does not present any significant challenges in terms of its application.

## Author contributions

TW: Writing – original draft. QZ: Supervision, Writing – review & editing, Writing – original draft. YQ: Writing – review & editing. YJ: Supervision, Writing – review & editing. FX: Supervision, Writing – review & editing. JD: Writing – review & editing. XZ: Resources, Writing – review & editing.
